# Array comparative genomic hybridization in prenatal diagnosis of first trimester pregnancies at high risk for chromosomal anomalies

**DOI:** 10.1186/1755-8166-5-38

**Published:** 2012-09-17

**Authors:** Isabel Filges, Anjeung Kang, Vanessa Klug, Friedel Wenzel, Karl Heinimann, Sevgi Tercanli, Peter Miny

**Affiliations:** 1 Dr. med. Isabel Filges, Division of Medical Genetics, University Children’s Hospital and Department of Biomedicine, University of Basel, Burgfelderstrasse 101, Building J, CH-4055, Basel, Switzerland; 2 Ultrasound Unit, Department of Obstetrics and Gynecology, University Hospital, Basel, Switzerland; 3 Department of Medical Genetics, BC Children’s and Women’s Hospital, Child and Family Research Institute, University of British Columbia, Box 153, 4480 Oak Street, Vancouver, BC V6H 3V4, Canada

**Keywords:** Prenatal diagnosis, Array comparative genomic hybridization, ArrayCGH, aCGH, First trimester, High risk, CVS

## Abstract

**Objective:**

To describe the diagnostic performance of array comparative genomic hybridization (aCGH) as a potential first line diagnostic method in first trimester high risk pregnancies.

**Method:**

In a retrospective study we performed aCGH using a targeted array BAC platform (Constitutional Chip® 4.0, PerkinElmer, Turku Finland, median resolution 600 kB) and the Affymetrix Cytogenetics® Whole Genome 2.7 M array (at a resolution of 400kB) on 100 anonymized prenatal samples from first trimester high risk pregnancies with normal conventional karyotype. We studied the technical feasibility and turn-around-time as well as the detection rate of pathogenic submicroscopic chromosome anomalies and CNVs of unknown significance.

**Results:**

We obtained results in 98 of 100 samples in 3 to a maximum of 5 days after DNA extraction. At the given resolution we did not identify any additional pathogenic CNVs but two CNVs of unknown significance in the chromosomal regions 1q21.1q21.2 (deletion) and 5p15.33 (duplication) (2%).

**Conclusion:**

In accordance with a growing number of reports this study supports the concept that aCGH at a resolution of 400-600kB may be used as a first line prenatal diagnostic test with high diagnostic safety and rapid turn-around time in high-risk first trimester pregnancies. Detection rate of CNVs of unknown significance, considered as a major hindrance for replacing conventional karyotyping by aCGH, is 2%, but the diagnosis of additional submicroscopic anomalies in this heterogeneous group of patients seems to be rare.

## Background

The current approach to prenatal diagnosis of chromosomal anomalies is a non-invasive risk assessment for trisomy 21 and other aneuploidies by first trimester or integrated risk screening offered to all pregnant women. If these screening approaches reveal an increased risk standard chromosome analysis after chorion villous sampling (CVS) or amniocentesis (AC) is offered for the detection of numerical or structural chromosome aberrations.

The current approach of risk screening has significantly reduced the number of invasive procedures and has increased the detection rate at the same time [1,2]. Recently, detection of aneuploidy by using massively parallel sequencing approaches as well as fetal-specific DNA-methylation ratio on free fetal DNA in maternal circulation (so-called non-invasive prenatal diagnosis) has been successful and appears to have the potential to replace first trimester screening in the near future [3-8].

In clinical practice, however, pregnancies at high risk for aneuploidy for various reasons do not infrequently show a normal conventional chromosome analysis. The limited clinical information provided by the ultrasound image of the fetal anatomy and physical development does usually not allow the diagnosis of a specific disease to be confirmed by appropriate testing which leaves parents in a dilemma of accepting a high risk for a condition with developmental delay and/or mental retardation of unknown severity or opting for a termination of the pregnancy.

Array comparative genomic hybridization (aCGH), which can provide a much higher resolution than conventional karyotyping, has now become the genetic test of first choice for postnatal investigation of intellectual disability (ID) and/or multiple congenital anomalies (MCA) detecting causal submicroscopic chromosomal imbalances i.e. microdeletion- and microduplication syndromes in 10-20% in these groups of patients [9,10]. A limited number of prenatal studies using array based methods have been conducted on fetuses with normal karyotypes focusing on high risk for aneuploidy defined by ultrasound anomalies [11-14] showing variable detection rates superior to conventional cytogenetics. Still, important questions are unanswered as to the appropriate platform and resolution, clinical indications, detection rate for pathogenic imbalances and copy number variations with unknown significance as well as cost-benefit and standardisation aspects in particular to the necessary follow-up studies by conventional karyotyping and/or fluorescence-in-situ hybridization (FISH) [15,16]. In particular, CNVs of unknown clinical significance are considered to be the major hindrance of introducing array-based technologies into prenatal diagnosis as a first-line approach. Therefore at present, array diagnosis has only been proposed as an adjunct tool in the investigation of pregnancies with congenital anomalies and normal karyotype [17].

The significant advances in earlier screening and diagnostic testing for genetic disorders have shifted prenatal diagnosis from screening and diagnosis form the second trimester to the first trimester. In experienced tertiary ultrasound centers, the detection rate of fetal structural anomalies is as high as 40% in the first trimester ultrasound [18]. As first trimester screening aims to provide early diagnosis we consider chorionic villi sampling as the invasive method of choice. Array-based methods have the potential to significantly reduce the turn-around time and replace rapid testing by interphase FISH, QF-PCR or MLPA. aCGH on chorionic villi could offer a time-saving and early approach to a comprehensive and high resolution assessment of chromosomal abnormalities following first trimester risk screening.

Between May 2009 and January 2011 we conducted a retrospective study in our tertiary antenatal referral center with a high proportion of high risk pregnancies applying array analysis for pregnancies at high risk for chromosomal anomalies in the first trimester. As a standard of care in our center CVS is offered to women with a confirmed high risk first trimester pregnancy allowing a comprehensive low resolution karyotype in a 24 h direct preparation followed by a conventional karyotype from cultured cells. Amniocentesis for the same indication is performed when CVS cannot be considered for obstetrical reasons or because of patients’ personal preferences. We used aCGH on anonymized cryopreserved stored or remaining tissue from prenatal invasive testing from pregnancies at a high risk for chromosomal anomalies and normal karyotype. Details on analyzed tissues are given in Table 1. High risk was defined as a high risk in first trimester screening (1:10 or higher) or nuchal translucency > 3 mm or both, intrauterine growth retardation and/or fetal malformation(s). Data on the clinical indication for prenatal invasive diagnosis, karyotype result, tissue and DNA quality as well as results of aCGH analysis including pathogenic CNVs not detected by conventional chromosome analysis as well as CNVs of unknown significance were collected.

**Table 1:** Distribution of sample types used in this study

	**Tissue**	**Number of samples**
CVS	Stored	13
	cryopreserved	
	Direct villi	23
	Cultures villi	50
Amniocentesis	Cultured amniocytes	13
others	Pleural liquid	1
**Total**		**100**

The goal of our study was to optimize technical aspects and to evaluate technical feasibility, safety and the potential of automation of aCGH in first trimester prenatal diagnosis as a first line test with the ultimate goal to replace direct preparation of chorionic villi that is used for rapid testing in our laboratory as well as other rapid aneuploidy testing methods. In addition detection rates of additional pathogenic variants compared to the rate of undesirable variants of unknown significance were assessed.

## Results

After technical optimization of DNA sample quality two analyzed samples (2%) failed to give interpretable results. In general, DNA quality of cryopreserved cell cultures, depending also on the period of storage, was lower than DNA quality of fresh tissue. Preferably, DNA extracted from direct chorionic tissue was used for analysis. However, DNA from cultured cells was used when direct villi were either not available any more or its total amount was insufficient for additional array analysis. In our retrospective cohort defined by first trimester high risk for chromosomal anomalies and normal conventional chromosome analysis we did not identify any single additional clear-cut chromosomal anomaly by using aCGH. Detection rate of CNVs of unknown significance was 2%. One deletion was found in the chromosomal region 1q21.1q21.2 (CVS sample) not overlapping with the recurrent 1q21.1 deletion syndrome and another CNV (CVS sample) was identified in the cri-du-chat critical region, but represented a duplication to which thus far no phenotype is attributed (Table 2). aCGH provided additional information on the extent of the aberrations for two CVS samples for which conventional karyotyping had revealed structural chromosomal anomalies. In one fetus with malformations additional material on the long arm of chromosome 20q turned out to be a complete trisomy of 20q with final karyotype designation 46,XY,der(20)(20pter- > 20qter::20q11.21- > 20q13.33).arr  20q11.21q13.33(20,066,572-49,655,888)x3. A pregnancy with high risk in first trimester screening but normal ultrasound was diagnosed with a terminal deletion 9p encompassing the region 9p24.3p24.2 (199,111-4,401,383; hg18) and a duplication of 2p25.3p24.3 (19,477-14,322,431; hg18) allowing more precise genotype-phenotype correlation particularly for the terminal 9p deletion. The extent explained a normal ultrasound but implies a high risk for a global developmental delay [19] and thus allows a more reliable outcome prediction.

**Table 2:** Copy number variants of unknown significance

**CNV Nr.**	**Type of aberration**	**BAC Clone-ID**	**Genome position Hg18**	**Maximal size of aberration**	**Chromosomal region**	**Interpretation**
		**Start**				
		**End**				
1	Deletion (2 clones)	RP5-998N21	147,596,844-147,726,269	0.568 Mb	1q21.1q21.2	Unknown significance, Not overlapping with the recurrent deletion in the 1q21.1 syndrome
		RP11-196G18	147,976,465-148,165,420			
2	Duplication (4 clones)	RP11-20B3	3,055,343-3,213,642	0.656 Mb	5p15.33	Unknown significance, Region deleted in cri-du-chat syndrome
		CTD-2012M11	3,558,697-3,712,084			

We encountered 3 remarkable situations with mosaicism of the fetoplacental unit. The specific issue of placental mosaicism and its consequences for the diagnostic accuracy have been addressed separately [20].

## Discussion

In our retrospective study we failed to show that the detection rate of aCGH at a median resolution of 600 kb (400 kb respectively) is superior to conventional chromosome analysis. The detection rate of CNVs of unknown significance, discussed as a major hindrance to the introduction of aCGH in prenatal diagnosis, is at 2%. Due to the retrospective anonymous design of the study we were not able to confirm or exclude familial segregation to further elucidate pathogenicity.

In contrast to the existing reports focusing on detection rates in pregnancies with fetal malformations mostly diagnosed by second trimester sonography we dealt with pregnancies showing a high risk for chromosomal anomalies in the first trimester for heterogeneous clinical reasons. Clinical indications for invasive prenatal testing were increased nuchal translucency (>3 mm) and/or elevated first trimester test risk in 57, more than a half, of the referred samples. Recently, Leung et al. reported on the detection of 4 additional pathogenic submicroscopic anomalies ranging from 1.2 to 7.9 Mb when using a custom designed array at a median resolution of 100 kb in a series of 48 pregnancies with nuchal translucency more than 3.5 mm [21]. However, 2 of these fetuses had additional sonographic anomalies. Both sample sizes, Leung’s and our’s, are comparably small and the overall detection rate of pathogenic CNVs in fetuses with increased NT seems to be rather low, but might nevertheless require further and larger studies. The progression or regression of cystic hygroma should probably be taken into account for phenotype correlations, and other monogenic disorders such as Noonan or CFC -syndrome caused by mutations in genes of the RAS-signalling pathway, seem to be a reasonable differential diagnosis to consider [22-24] (and personal communication Yntema H.G. European Society Human Genetics C07.6).

In our approach we have chosen a comparably low and targeted resolution of a median 600 kb resolution for the BAC-array in order to avoid CNVs of unknown clinical significance. In a prenatal setting unknown CNVs can represent a particular challenge as phenotypic data are sparse compared to the possibilities of obtaining phenotypic details on postnatal patients. In addition, the potential option of pregnancy termination requires a higher level of interpretation accuracy. Our resolution might be too cautious especially considering our low detection rate of pathogenic imbalances. Most postnatally identified pathogenic microdeletion- and duplication syndromes, however, are caused by pathogenic CNVs larger than 400 kb [25]. Therefore, a whole genome resolution of 400 kb should provide a reliable prenatal diagnosis. After extensive individual counselling diagnostic array analysis is now offered as a diagnostic choice in high risk pregnancies with fetal malformations and normal conventional chromosome analysis lacking further diagnostic options or to determine the precise nature of a structural aberration detected by conventional karyotyping.

D’Amours et al. investigated a series of 49 fetuses with ultrasound anomalies using custom-designed whole genome oligo-arrays with different resolutions [26]. Out of 4 clinically significant anomalies, all detected by high resolution arrays, only 1 was smaller than 400 kb, one was about 700 kb and 2 were potentially detectable by conventional chromosome analysis at sizes of about 8 Mb and 14 Mb. Recent high resolution array studies using heterogeneous array designs including BAC-, oligo- and SNP arrays also focusing on detection rates in fetuses with congenital malformations [12,27-29] yielded variable detection rates of pathogenic CNVs in 1–3% up to 10% [13], and even up to 16% [14]. However, sizes of the clinically relevant aberrations were larger than 1 Mb [12,28,29]. Even in the studies using whole genome SNP-arrays with a resolution higher than 100 kb clinically relevant aberrations were mainly in the range of 1 Mb or more [13] or larger than 500 kb or 1 Mb [14,25] in all but one case [14]. An overall detection rate of 5-6% also including chromosomal anomalies detectable by conventional karyotyping was shown in array studies dealing with samples of heterogeneous clinical indications including fetal structural anomalies [12,30,31]. Hillman et al. concludes an overall detection rate of 3.6% regardless of the clinical indication and 5.3% for cases with ultrasound anomalies in his review and meta-analysis of this recent heterogeneous literature [32].

Referring to these results we would have expected to find at least one additional submicrosopic anomaly in our cohort examined. We focused, however, on pregnancies at risk for chromosomal anomalies as determined by first trimester screening, which has not been systematically considered in previous studies. Our patient cohort likely represents heterogeneous clinical indications, the main group being referred for increased nuchal translucency and elevated first trimester risk screening, whereas the proportion of fetuses with congenital malformations is comparatively low. Our results are in accordance with the findings of Faas et al. (personal communication, ESHG 2011 P05.32) who did not find any clinically relevant abnormalities in a cohort of 95 fetal samples with ultrasound anomalies including increased nuchal translucency when implementing a strategy for routine array diagnosis in parallel to QF-PCR. High resolution whole genome arrays may therefore be specifically indicated in pregnancies with distinct fetal ultrasound anomalies or in the precise determination of rare structural chromosomal anomalies.

Our approach revealed a detection rate of 2% for CNVs of unknown significance at the given resolution, confirming that aCGH seems to be a safe approach in the diagnosis of first trimester pregnancies considered at high risk for chromosomal anomalies. In our experience aCGH is technically feasible and reliable as a first-line diagnostic test for prenatal samples, replacing laborious direct preparation of CVS as well as other rapid but less comprehensive testing procedures. We were able to provide a result in 3 to a maximum of 5 days after DNA extraction offering a superior coverage of pathogenic aberrations in the genome. Thus, aCGH provides the advantage to give patients a more complete genome analysis result in a short turn-around-time. High resolution arrays may gain further importance as we can expect that the clinical use of non-invasive diagnosis for common aneuploidies will increase the proportion of samples from high risk pregnancies obtained by invasive diagnosis. However, the appropriate array resolution still remains a matter of debate and may need to be adapted to the specific clinical indication and/or parental expectations. These would require a flexible resolution design of arrays and analysis software as well as the possibility to analyze one single patient in a (very) short turnaround time or multiple patients in parallel for diagnostic laboratories. Platforms requiring small initial DNA amount will be advantageous as the DNA amount from direct villi or amniocytes will be sufficient to allow array analysis as compared to time-consuming cell culturing for DNA extraction. For the time being cell cultures may be used as a back-up for confirmational FISH-analysis as well as follow-up of mosaic results if aCGH reveals an unbalanced structural aberration.

## Conclusion

For the future application of aCGH in prenatal diagnosis we see the potential to use aCGH at a resolution of 400–600 Kb as a first line genetic test including the replacement of rapid testing methods such as direct preparation of chorionic villi, interphase-FISH and DNA-based QF-PCR and MLPA with a high diagnostic safety and accuracy. Considering results of recent studies high resolution arrays may be specifically indicated for pregancies with fetal malformations. So far, prenatal conventional chromosome analysis has been a rather standardized procedure, but array diagnosis may put pressure on prenatal professionals to think about personalizing diagnostic approaches.

## Material and Methods

### Ethics approval

The study was approved by the local ethics committee EKBB (Ref.Nr.EK: 62/08).

### Samples

After technical optimization and validation of aCGH platforms by using anonymized samples with known chromosomal aberrations, we performed aCGH analysis of so far 100 prenatal samples from high risk pregnancies as defined in the study design. Of 100 patients 88 samples were included in the anonymized retrospective study. 12 patients seeked diagnostic testing, 2 of which had structural anomalies in conventional karyotyping, which were characterized more precisely by array, and 3 had mosaic results on which we reported in detail previously [20]. A survey of clinical indications is given in Table 3.

**Table 3:** Clinical indications for invasive prenatal diagnosis in our retrospective cohort including 12 diagnostic samples

**Indication**	**Number of samples**	**% of total number of samples**
NT>3 mm	26	26.5
Risk of 1:10 or higher in first trimester screening	9	9.2
Both (NT>3mm and risk > 1:10)	22	22.5
**Subtotal**	57*	**58.2**
IUGR	10	10.2
IUGR and malformation	10	10.2
Malformation(s)	21**	21.4
**Total**	98***	**100**

* investigation of 2 mosaic and 1 chromosomal structural anomaly included

** investigation of 1 mosaic result and 1 chromosomal structural anomaly included.

*** 2 samples failed analysis.

### Conventional chromosome analysis

Conventional chromosome analysis was done on chorionic villi according to standard procedures evaluating numerical chromosome aberrations in direct preparation of cytotrophoblastic cells (QFQ-banding) and long-term culture of mesenchymal tissue for numerical and structural aberrations (GTG-banding, 500 band level). Direct preparation of chorionic villi allows the separation and analysis of cells from the mitotically active cytotrophoblastic layer after 24 hours. Chromosome analysis on amniotic fluid was performed according to standard protocols.

### DNA extraction

The fetal sample was prepared for aCGH analyses using either direct genomic DNA from chorionic villi, DNA obtained from cultured CVS cells or DNA from amniocyte cultures. DNA for subsequent aCGH was extracted from 10–20 mg of direct chorionic villi or a T75 flask cell culture using the Qiagen DNeasy Blood and Tissue kit (Qiagen Germany Gmbh, Hilden) following the manufacturer’s instructions. DNA from unprocessed and uncultured direct villi represents a mixture of cytotrophoblastic and mesenchymal cell sources whereas DNA from cultured cells is of mesenchymal origin. Due to experiences when studying aCGH in the presence of fetoplacental mosaicism [20] initial lysis of chorionic villi by proteinase K was followed by collagenase digestion to increase the proportion of DNA from mesenchymal cells. RNase treatment (RNase A, Qiagen Germany Gmbh, Hilden) was added to remove the high levels of RNA in these tissue types. DNA concentration and purity was determined using a NanoDrop 1000 spectrophotometer (Witec AG, Littau, Switzerland).

### Array comparative genomic hybridization (aCGH)

aCGH was performed on extracted DNA in 95 study samples using the Constitutional Chip® 4.0 (PerkinElmer, Turku Finland) BAC platform. Validation analysis was done on 20 samples with known chromosomal aberrations. The BAC array contains more than 5200 distinct clones in total with a median probe spacing of 600 kb. Targeted regions with higher clone density are chromosomal regions of interest (>1200 clones), subtelomeric regions with coverage by more than 900 BAC clones, pericentromeric regions with enhanced coverage (>100 clones) and the X-chromosome with near to tiling coverage for high resolution detection of chromosomal abnormalities across the X-chromosome. An additional back-bone of more than 2000 BAC clones provides an average resolution of less than 1 Mb in non-targeted areas. Two 2 μg of purified DNA is required for analysis. Labelling and hybridization of test and reference DNA was performed according to the manufacturer’s protocols. Test and reference DNA were labelled by Cy3 and Cy5 with reciprocal labelling reactions and hybridized for 16–18 hours on two independent arrays (dye-swap) which allows to normalize intensity bias introduced by different dye properties. Arrays were then subjected to 4 washings according to the protocol. Two colour scanning was performed by the PerkinElmer® Inc.'s ScanArray® Microarray Scanner. Acquisition of the microarray images was performed with the ScanArray Express software, Microarray Analysis System, version 4.0.0.0004. Data extraction, analysis and visualisation were done by the Oneclick CGH software, version 4.3.3., Perkin Elmer Edition. Classification of gain and loss was based on the software’s segmentation algorithm with a threshold of a 1.2 ratio (0.26 log2 ratio). A minimum of two consecutive clones indicating copy number variation was considered as a relevant aberration.

Due to a technology change in our laboratory the Affymetrix Cytogenetics® Whole Genome 2.7 M array, which is based on 2.7 million copy number markers (CN markers) and additional 400,000 SNPs (average marker spacing 1086 bp) was used in further 5 samples for diagnostic purposes. We use this platform for the postnatal investigation of patients with syndromic and non-syndromic intellectual disability with an overall detection rate of pathogenic CNVs of 12.2% in patients with previously normal karyotype [33]. Validation for prenatal tissues was performed on 16 control samples. Only 150 ng of purified DNA is required for analysis which represents a significant advantage over the 2 μg required for the BAC array when using prenatal tissues. Single colour hybridization was performed according to the manufacturer`s protocol. Arrays were then scanned by the GeneChip Scanner 3000 7 G system (Affymetrix Inc. Santa Clara, CA, USA). Results were analyzed by the Affymetrix Chromosome Analysis Suite (ChAS®) software. Aberrations were filtered by the software up to a minimal size of 400 kB for deletions and duplications. Limits were decided based on our own previous experiences and existing literature. Results were reassessed manually for CNVs.

### Fluorescence-in-situ hybridization (FISH)

FISH was used for confirmation of dosage imbalances suspected to be pathogenic in the samples when possible on remaining tissue culture. Confirmation or exclusion of involvement in familial rearrangements in parental samples was not realizable due to the retrospective design of the study. FISH was performed on metaphase slides according to the manufacturer’s hybridization protocols. Clones from Abbott and Kreatech were used when available for the aberrant region and for control hybridization. Clones provided by BlueGnome were chosen within specific regions when the former were not available.

### Interpretation of CNV significance

The pathogenicity of the CNVs was assessed using the guidelines recently discussed [34-37]. All detected CNVs were compared against the database of genomic variants (http://projects.tcag.ca/variation). If an aberration was not reported as a benign variant, it was, in correlation with the phenotype, analyzed for gene content, function and genomic position potentially in relation to the phenotype or a particular phenotypic trait, compared with entries in the Decipher database (http://decipher.sanger.ac.uk) or Ecaruca (http://www.ecaruca.net) and evaluated against existing literature for known syndromes and overlapping causal aberrations. Aberrations likely to be causative were confirmed by FISH. Aberrations of unknown significance were documented, however, parental array analysis was not possible for samples included in the retrospective testing.

## Abbreviations

aCGH = Array comparative genomic hybridization; CNV = Copy number variant; CVS = Chorionic villous sampling; AC = Amniocentesis; ID = Intellectual disability; MCA = Multiple congenital anomalies; FISH = Fluorescence-in-situ-hybridization.

## Competing interests

All authors declare no competing interests.

## Author’s contributions

IF and PM designed and coordinated the study and wrote the manuscript. IF collected and interpreted clinical and array data. AK and ST are responsible for prenatal ultrasound and invasive procedures and participated in patient recruitment. VK performed the BAC-arrays. FW, IF, KH and PM are responsible for array interpretation. All authors read and approved the final manuscript.

## Details of ethics approval

The study was approved by the local ethics committee EKBB (Ref.Nr.EK: 62/08). For diagnostic testing individual patients’ consent was obtained within the counselling process.
